# Immunization of Rabbits with Recombinant Human Cytomegalovirus Trimeric versus Monomeric gH/gL Protein Elicits Markedly Higher Titers of Antibody and Neutralization Activity

**DOI:** 10.3390/ijms20133158

**Published:** 2019-06-28

**Authors:** Xinle Cui, Zhouhong Cao, Shuishu Wang, Michael Flora, Stuart P. Adler, Michael A. McVoy, Clifford M. Snapper

**Affiliations:** 1 Department of Pathology, Uniformed Services University of the Health Sciences, Bethesda, MD 20814, USA; 2 Department of Biochemistry, Uniformed Services University of the Health Sciences, Bethesda, MD 20814, USA; 3 Department of Anatomy, Physiology and Genetics, Uniformed Services University of the Health Sciences, Bethesda, MD 20814, USA; 4 CMV Research Foundation, Richmond, VA 23229, USA; 5 Department of Pediatrics, Virginia Commonwealth University, Richmond, VA 23298, USA

**Keywords:** human cytomegalovirus (HCMV), vaccine, HCMV gB, HCMV gH/gL, core fusion machinery, neutralizing antibody, congenital infection, solid organ transplantation, hematopoietic stem cell transplantation

## Abstract

Congenital human cytomegalovirus (HCMV) infection and HCMV infection of immunosuppressed patients cause significant morbidity and mortality, and vaccine development against HCMV is a major public health priority. HCMV envelope glycoproteins gB, gH, and gL, which constitute the core fusion machinery, play critical roles in HCMV fusion and entry into host cells. HCMV gB and gH/gL have been reported to elicit potent neutralizing antibodies. Recently, the gB/gH/gL complex was identified in the envelope of HCMV virions, and 16–50% of the total gH/gL bound to gB, forming the gB/gH/gL complex. These findings make the gB/gH/gL a unique HCMV vaccine candidate. We previously reported the production of HCMV trimeric gB and gH/gL heterodimers, and immunization with a combination of trimeric gB and gH/gL heterodimers elicited strong synergistic HCMV-neutralizing activity. To further improve the immunogenicity of gH/gL, we produced trimeric gH/gL. Rabbits immunized with HCMV trimeric gH/gL induced up to 38-fold higher serum titers of gH/gL-specific IgG relative to HCMV monomeric gH/gL, and elicited ~10-fold higher titers of complement-dependent and complement-independent HCMV-neutralizing activity for both epithelial cells and fibroblasts. HCMV trimeric gH/gL in combination with HCMV trimeric gB would be a novel promising HCMV vaccine candidate that could induce highly potent neutralizing activities.

## 1. Introduction

Human cytomegalovirus (HCMV) is an enveloped, double-stranded DNA β-herpesvirus of the Herpesviridae family. HCMV causes infection in 40% to 60% of the population in industrialized countries and 80–100% of the population in developing countries [1,2]. Congenital HCMV infection of neonates and the infection of transplant recipients and patients with HIV/AIDS cause significant morbidity and mortality, though HCMV infection in immunocompetent individuals is generally asymptomatic [1,2,3,4,5]. Congenital HCMV infection is the leading non-genetic cause of hearing loss in childhood, and it is the most common infectious cause of brain damage in developed countries [1,2,6,7,8]. Additional diseases caused by congenital HCMV infection include chorioretinitis resulting in vision loss, hepatitis, intracranial calcifications, seizures, cerebral palsy, microcephaly and neurodevelopmental delay [1,2,6,7]. HCMV infection causes end-organ diseases such as hepatitis and pneumonitis in solid organ and hematopoietic stem cell transplant patients, and HCMV viremia could significantly increase the chance of graft rejection, graft failure, and in hematopoietic stem cell transplant patients, graft-versus-host disease [9,10,11,12]. The incidence of HCMV infection remains high despite active monitoring and management with antiviral drugs, ranging from 20% to 70% in the first year post-transplantation, and in solid organ and hematopoietic stem cell transplant recipients, HCMV infection remains one of the most common complications affecting patient survival [13,14,15,16,17]. 

HCMV is spread horizontally via saliva and urine, and transplacentally to the fetus [18,19]. The target cells of HCMV include epithelial cells, endothelial cells, monocyte-macrophages, hepatocytes, fibroblasts, and neurons [20,21,22]. HCMV enters cells by fusing its envelope with either the plasma membrane or endosomal membrane, which is a mechanism that is analogous to that employed by other members of the herpesvirus family [20,21,22,23]. HCMV envelopes glycoproteins gB, gH, gL, and gO, as well as the UL128, UL130, and UL131A proteins have collectively been identified as playing critical roles in HCMV fusion and entry into host cells [20,22]. The gB protein is the direct mediator of HCMV fusion with all the host cell membranes, and the activation of HCMV gB requires its association with the gH/gL/gO protein complex [24,25]. For the HCMV infection of epithelial and endothelial cells, an alternative protein complex consisting of gH/gL with UL128, UL130, and UL131A (the pentameric complex) is further required for efficient targeting [20,23,26,27].

There is currently no HCMV vaccine approved for clinical use, although the development of an HCMV vaccine is a public heath priority. Over the past 50 years, a variety of experimental vaccine approaches have been evaluated, and many are currently in various stages of evaluation [28,29,30,31]. An HCMV vaccine consisting of an adjuvanted recombinant monomeric gB protein has advanced the furthest in clinical trials [32,33]. Specifically, several phase I and phase II clinical trials utilizing a recombinant monomeric HCMV gB in microfluidized adjuvant 59 (MF59) have been completed and demonstrated approximately 50% efficacy in the prevention of HCMV infection [19,34,35,36,37]. The recombinant gB used in these clinical trials was originally developed at Chiron Corporation (Chiron gB), and was expressed as a truncated, secreted polypeptide [38]. The Chiron gB did not recapitulate the trimeric conformation of native gB expressed on the envelope of virions or on the surface of HCMV-infected cells, and therefore, recombinant gB proteins expressing conformational epitopes could potentially elicit broader and highly efficient vaccine responses [38,39]. 

We previously reported the production of a HCMV trimeric gB by the insertion of a flexible 15 amino acid (Gly_4_Ser)_3_ linker at the furin cleavage site that allowed for terminal protein folding and efficient expression [40]. HCMV trimeric gB induced significantly higher serum titers of gB-specific IgG relative to a HCMV monomeric gB similar to the Chiron gB, and elicited markedly higher complement-dependent and complement-independent HCMV-neutralizing activities [40]. More importantly, compared to the monomeric gB, the trimeric gB elicited markedly higher cross-strain neutralization activities against several clinical HCMV strains as well as a variant of strain AD169 (AD169^wt131^) that expresses a functional pentameric complex [40]. 

We recently reported that immunization with the combination of HCMV envelope proteins trimeric gB, gH/gL, and/or UL128/UL130/UL131A elicited strong synergistic HCMV-neutralizing activities, and predicted that the combination of trimeric gB with gH/gL or the pentameric complex might be ideal HCMV vaccine candidates [41]. The discovery that in the envelope of HCMV virions, up to 50% of gH/gL complexed with gB makes the concept of a vaccine comprised of trimeric gB with gH/gL even more attractive [42]. However, since immunization with recombinant HCMV monomeric gH/gL only elicited moderate neutralizing activity, the trimerization of the protein would substantially increase its overall immunogenicity, including the induction of neutralizing antibodies, as described generally for protein multimerization [43,44,45,46,47]. The use of a combination of HCMV trimeric gB with trimeric gH/gL could significantly increase the efficacy of the HCMV vaccine candidate [41].

## 2. Results

### 2.1. Production of Monomeric and Trimeric HCMV gH/gL Recombinant Proteins

**Figure 1:**
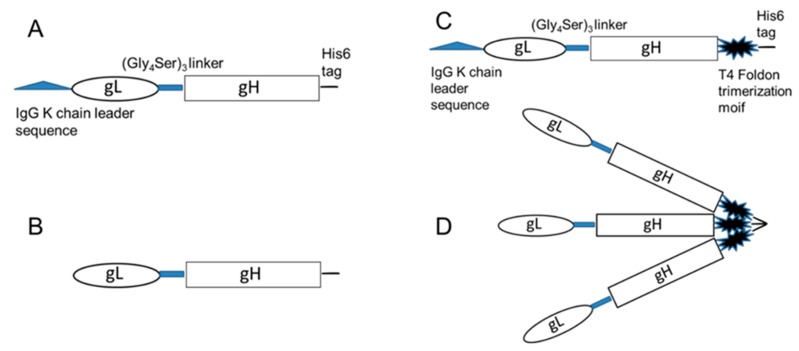
DNA constructs encoding human cytomegalovirus (HCMV) monomeric and trimeric gH/gL. (**A**) Diagram of the DNA sequences for production of monomeric HCMV gH/gL. (**B**) Diagram of recombinant HCMV monomeric gH/gL protein. (**C**) Diagram of the DNA sequences for the production of HCMV trimeric gH/gL. (**D**) Diagram of recombinant HCMV trimeric gH/gL protein.

We previously designed DNA constructs to produce in Chinese hamster ovary cells (CHO) the recombinant HCMV trimeric gB protein as well as the recombinant gB, gH/gL, and gp350 proteins of Epstein Barr virus (EBV), which is another member of the human herpesviruses family [40,43,47]. In the current study, we took a similar approach to design DNA constructs that encode for either monomeric or trimeric HCMV gH/gL proteins (Figure 1). A 5’ end IgG κ leader sequence was introduced to promote protein secretion, followed by sequences encoding HCMV gL and gH with a (Gly_4_Ser)_3_ linker in between to allow for the proper folding of both proteins (Figure 1A–D). HCMV gL functions as a chaperon protein for gH, and the expression of the gL protein first, followed by gH, would ensure the correct folding of the gH protein. The foldon trimerization domain coding sequence derived from T4 phage fibritin would promote the gH/gL trimer folding into its terminal form (Figure 1C,D). A His_6_ tag sequence was added to the 3’ end of the DNA constructs encoding for recombinant proteins to allow for efficient purification.

**Figure 2:**
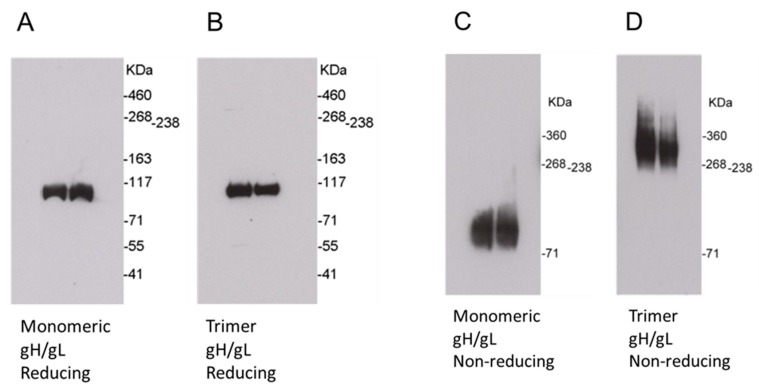
Western blot analysis of HCMV monomeric and trimeric gH/gL recombinant proteins under reducing and non-reducing conditions. Secreted proteins were purified with affinity purification and size exclusion chromatography from culture supernatants of stable Chinese hamster ovary (CHO) cell lines. (**A**) and (**B**) Blots of HCMV monomeric and trimeric gH/gL recombinant proteins developed with an anti-HCMV gH monoclonal antibody under reducing conditions. (**C**) and (**D**) Blots of HCMV monomeric and trimeric gH/gL recombinant proteins developed with an anti-HCMV gH monoclonal antibody under non-reducing conditions.

Synthesized DNA coding for monomeric or trimeric gH/gL was cloned into pOptiVEV (Thermo Fisher Scientific, Waltham, MA, USA) and transfected CHO cells. Following selection with increasing concentrations of methotrexate, stable gH/gL expressing CHO cell lines were generated by limiting dilution cloning. Then, CHO cells were cultured in a FiberCell bioreactor (FiberCell Systems, Frederick, MD, USA). The supernatants were concentrated for affinity purification using a cobalt column (Thermo Fisher Scientific, Waltham, MA, USA), and further purified using size exclusion chromatography on a Superdex 200 column (GE Lifesciences, Pittsburgh, PA, USA). Western blot analysis of the monomeric gH/gL protein using an anti-HCMV gH monoclonal antibody under either reducing conditions or modified non-reducing conditions demonstrated a single size band of ~110 kDa, which is consistent with gH/gL heterodimers (Figure 2A,C). Western blot analysis of the trimeric gH/gL protein under reducing conditions disrupted the trimeric structure, and showed a single size band of ~110 kDa (Figure 2B). Under modified non-reducing conditions, Western blot analysis of the trimeric gH/gL protein demonstrated a single size band of ~330 kDa, which is consistent with a trimer of the gH/gL heterodimer (Figure 2D).

### 2.2. HCMV Trimeric gH/gL Protein Induced Markedly Higher Serum Titers of gH/gL-Specific IgG Relative to Monomeric gH/gL

**Figure 3:**
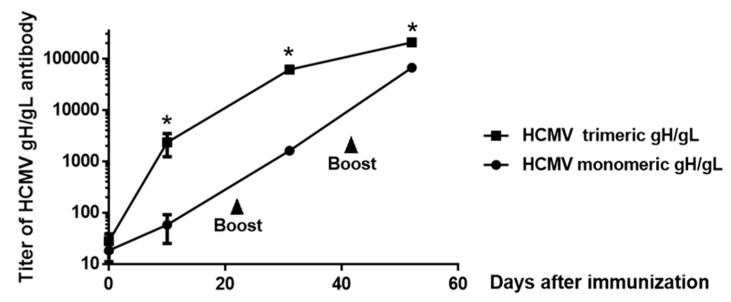
Serum titers of gH/gL-specific IgG following immunization with HCMV monomeric and trimeric gH/gL recombinant proteins. Groups of rabbits, 12–15 weeks old (*n* = 5) were immunized subcutaneously with 25 µg of HCMV monomeric versus trimeric gH/gL recombinant protein adjuvanted with alum + CpG-ODN, and boosted on days 21 and day 42 post-immunization. Sera were obtained 10 days following each immunization for the measurement of serum titers of gH/gL-specific IgG by ELISA. Significance * *p* < 0.05.

We directly compared HCMV monomeric and trimeric gH/gL for the elicitation of total serum titers of gH/gL-specific IgG. Groups of five adult rabbits each were immunized subcutaneously with 25 µg of HCMV monomeric or trimeric gH/gL recombinant protein adjuvanted with alum + CpG-ODN, and then boosted in a similar fashion on days 21 and 42 post-immunization. The CpG-ODN sequence was optimized for use in rabbits [48]. As illustrated in Figure 3, both the HCMV monomeric and trimeric gH/gL proteins induced augmented serum IgG responses following the first booster immunization, and further significant augmentation in serum IgG titers following the second booster immunization. HCMV trimeric gH/gL induced 29-fold and 38-fold higher serum gH/gL-specific IgG titers relative to monomeric gH/gL following the primary immunization and the first booster immunization respectively, with the difference of titers decreased to threefold after the second booster immunization (Figure 3). These data are consistent with our previous study in mice and rabbits using monomeric and multimeric EBV envelope proteins gp350 and gH/gL, in which the multimerization of proteins induced a marked increase in immunogenicity [43,47].

### 2.3. Immunization of Rabbits with HCMV Trimeric gH/gL Protein Elicited Significantly Higher HCMV-Neutralizing Antibodies for Epithelial Cells Relative to Monomeric gH/gL

**Figure 4:**
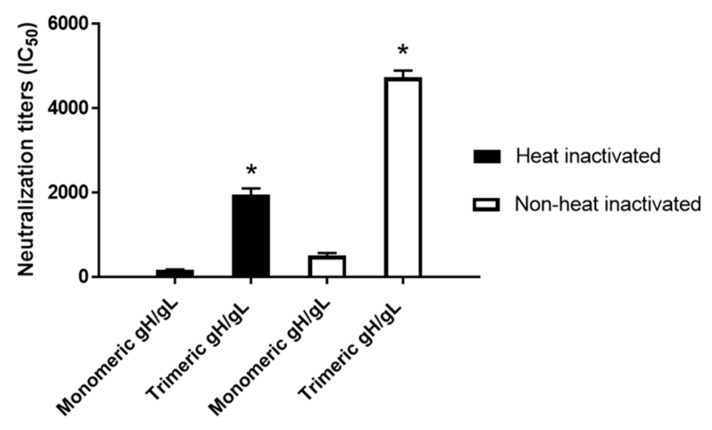
Immunization with HCMV trimeric gH/gL elicited significantly higher serum titers of complement-dependent and complement-independent HCMV-neutralizing activity for epithelial cells compared to monomeric gH/gL. Day 52 pooled sera from five rabbits immunized with 25 µg of HCMV trimeric versus monomeric gH/gL recombinant protein were heat-inactivated (closed bar) or non-heated (open bar), and IC50 neutralizing activity was determined of each pooled sample in quadruplicate using the ARPE-19 epithelial cell line and HCMV strain AD169^wt131^. Significance * *p* < 0.05 compared to the sera from rabbits immunized with HCMV monomeric gH/gL.

Multimeric proteins are known to be significantly more potent in the elicitation of virus neutralizing antibodies, as demonstrated by an EBV tetrameric gp350 [47]. Tetrameric gp350 elicited 20-fold to 40-fold higher EBV neutralizing antibodies compared to monomeric gp350 after immunization in mice [47]. In the current study, the HCMV-neutralizing antibodies of day 52 sera from the rabbits immunized with three doses of 25 µg each of HCMV trimeric versus monomeric gH/gL were analyzed using ARPE-19 epithelial cells and HCMV strain AD169^wt131^, the HCMV strain AD169 variant expressing a functional pentameric complex. Without heat inactivation to preserve the complement activity, sera from rabbits immunized with HCMV monomeric gH/gL showed moderate complement-dependent HCMV-neutralizing antibodies, and the sera from rabbits immunized with HCMV trimeric gH/gL demonstrated 11-fold higher complement-dependent HCMV-neutralizing antibodies relative to HCMV monomeric gH/gL (Figure 4). After heat inactivation at 56 °C for 30 min to inactivate the complement, the HCMV-neutralizing antibodies were both slightly decreased for the sera from rabbits immunized with HCMV monomeric and trimeric gH/gL. Compared to HCMV monomeric gH/gL, HCMV trimeric gH/gL elicited ninefold higher complement-independent HCMV-neutralizing antibodies for epithelial cells (Figure 4). 

### 2.4. HCMV Trimeric gH/gL Elicited Significantly Higher Serum Titers of HCMV-Neutralizing Activity for Fibroblasts Compared to Monomeric gH/gL

**Figure 5:**
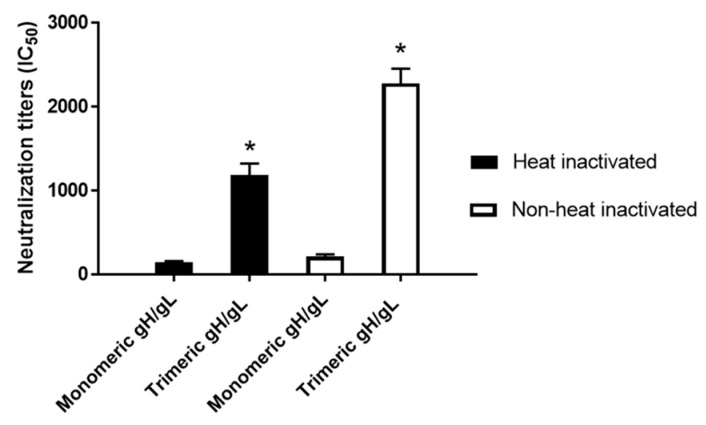
Immunization with HCMV trimeric gH/gL elicited significantly higher complement-dependent and complement-independent serum titers of HCMV-neutralizing activity for fibroblasts compared to monomeric gH/gL immunization. Day 52 pooled sera from five rabbits immunized with 25 µg of HCMV trimeric versus monomeric gH/gL were heat-inactivated (closed bar) or non-heated (open bar), and the IC50 neutralizing activity was determined of each pooled sample in quadruplicate using the MRC-5 fibroblast cell line and HCMV strain AD169^wt131^. Significance * *p* < 0.05 compared to the sera from mice immunized with HCMV monomeric gH/gL.

The HCMV-neutralizing antibodies of day 52 sera from the rabbits immunized with three doses of 25 µg each of HCMV trimeric versus monomeric gH/gL were also analyzed using MRC-5 fibroblasts and HCMV strain AD169^wt131^. Sera from trimeric gH/gL-immunized rabbits, in the absence of heat inactivation to preserve complement activity, demonstrated 10.5-fold higher serum titers of HCMV-neutralizing activity relative to sera from monomeric gH/gL-immunized rabbits (Figure 5). After heat inactivation at 56 °C for 30 min to inactivate the complement, the titers of HCMV-neutralizing activity slightly decreased for both groups, where the trimeric relative to the monomeric gH/gL group still demonstrated eightfold higher serum titers of HCMV-neutralizing activity (Figure 5).

## 3. Discussion

HCMV enters and infects target cells through the fusion of its envelope with either the plasma membrane or endosomal membrane, and requires multiple viral envelope proteins. Two distinct pathways have been identified as represented by the HCMV infection of fibroblast versus epithelial cells. The infection of fibroblasts involves HCMV envelope proteins gB, gH, gL, and gO, where gH, gL, and gO form a protein complex [20,21,22,23]. The gH/gL/gO protein complex interacts with the fibroblast cell membrane receptor PDGF-α, which then activates the gB protein for direct triggering of the virion envelope and cell membrane fusion through macropinocytosis [49,50,51,52,53]. The infection of epithelial cells with HCMV requires HCMV envelope proteins gB, gH, gL, and UL128/UL130/UL131A, where gH, gL, UL128/UL130/UL131A form a pentameric complex [20,23,26,27]. The pentameric complex binds to an epithelial cell receptor, neuropilin-2, which then triggers gB for endocytosis and fusion of the virion envelope with the endosomal membrane [53,54]. Although the gH/gL/gO complex is also implicated in HCMV infection of epithelial cells, its role is controversial, since epithelial cells express very low levels of PDGFα [53]. However, it is widely accepted that gB, gH, and gL—the components of the core fusion machinery—are required for infection of all the target cells with HCMV. 

As these HCMV envelope proteins play critical roles in HCMV infection and entry into target cells, they have been thoroughly studied as vaccine candidates against HCMV, especially gB and the pentameric complex. In a phase II study in postpartum seronegative women within one year of giving birth, the gB/MF59 vaccine demonstrated 50% efficacy against primary HCMV infection compared to women in the same cohort who received the placebo [19]. Another multi-center study in healthy HCMV-seronegative adolescent girls demonstrated 43% efficacy for the gB/MF59 vaccine in preventing primary HCMV infection, although the difference was not statistically significant [36]. The third phase II study of the gB/MF59 vaccine conducted in solid-organ transplant recipients demonstrated both a reduction in viremia and the total number of days requiring antiviral treatment compared to those who received placebo [37]. Further, the benefit of vaccination was most striking in HCMV-seronegative recipients of transplants from HCMV-seropositive donors, and the duration of viremia post-transplantation was inversely correlated with the magnitude of the gB antibody response [37]. The HCMV gB used in these clinical trials was Chiron gB, although this Chiron gB did not recapitulate the trimeric conformation of gB antigen expressed on HCMV virions or the surface of HCMV-infected cells, these Phase II clinical trials demonstrated ~50% efficacy in the prevention of HCMV infection, which is truly a milestone in HCMV vaccine development [28,29,30]. 

The immunogenicity of recombinant HCMV gB can be improved by the generation of a trimeric gB expressing native conformational epitopes. We have produced a HCMV trimeric gB by insertion of a flexible 15 amino acid (Gly_4_Ser)_3_ linker at the furin cleavage site that allowed for terminal protein folding and efficient expression [40]. Compared to a HCMV monomeric gB similar to the Chiron gB, trimeric gB induced up to 11-fold higher serum titers of gB-specific IgG, and elicited up to 50-fold higher complement-dependent and complement-independent serum HCMV neutralizing activities [40]. Importantly, compared to the monomeric gB, the trimeric gB elicited markedly higher cross-strain neutralizing activity against several clinical HCMV strains and AD169^wt131^ [40]. 

Many preclinical studies and clinical trials have also evaluated candidate vaccines incorporating the pentameric complex. A modified vaccinia virus Ankara (MVA) vectored vaccine expressing the pentameric complex elicited potent complement-independent and complement-dependent HCMV-neutralizing antibodies after the immunization of mice [55]. Mice as well as nonhuman primates immunized with lipid nanoparticles encapsulating modified mRNA encoding the HCMV pentameric complex elicited potent and durable neutralizing antibody titers [56]. A Phase I clinical trial of a replication defective (transgenic disabled infectious singlecycle, DISC) HCMV live attenuated vaccine expressing the pentameric complex demonstrated the induction of neutralizing antibody titers that were equal to or higher than HCMV seropositive subjects analyzed with epithelial cells [28,57].

Antibodies elicited by the pentameric complex are likely to prevent HCMV infecting epithelial cells, endothelial cells, and monocytes, but not fibroblasts or primary trophoblast progenitor cells [58,59,60,61,62,63,64,65]. Since HCMV gB elicits higher HCMV neutralization activity for fibroblasts than epithelial cells, whereas the pentameric complex mainly elicits high HCMV neutralization activity for epithelial cells, endothelial cells, and monocytes, it has been suggested that an optimal prophylactic HCMV vaccine will consist of both trimeric gB and pentameric complex proteins [31,66]. Immunization with the combination of gB and the pentameric complex has been tried using RNA vaccine or the MVA vectored vaccine expressing simultaneously gB and the five subunits of the pentameric complex, although enhanced HCMV-neutralizing activity was induced, no synergistic or additive effects in the elicitation of HCMV-neutralizing activity was reported [55,56]. We recently reported that immunization with the combination of recombinant HCMV trimeric gB, monomeric gH/gL, and UL128/UL130/UL131A induced strong synergistic HCMV-neutralizing activity for epithelial cells, and combined immunization with recombinant HCMV trimeric gB and monomeric gH/gL elicited strong synergistic HCMV-neutralizing activity for fibroblasts [41]. As expected, the addition of UL128/UL130/UL131A to gB and gH/gL did not further increase the serum HCMV-neutralizing activity elicited for fibroblasts, since UL128/UL130/UL131A is not required for the infection of these cells with HCMV [41]. 

In addition to the gH/gL/gO and pentameric complexes, another gH/gL protein complex was recently reported: the gH/gL/gB complex, or more appropriately the gB/gH/gL complex [42]. Since the core fusion machinery of HCMV consists of gB, gH, and gL, the identification of a preformed gB/gH/gL complex in HCMV virions is striking. The gB/gH/gL complex was previously thought only to exist transiently during membrane fusion after the gH/gL/gO complex or the pentameric complex binding to cell receptors and activating gB. More importantly, this study also showed that gB bound to gH/gL early after glycoprotein synthesis in the endoplasmic reticulum, and 16–50% of the total gH/gL bound to gB in the envelope of HCMV virions [42]. In contrast, the gH/gL/gO complex or the pentameric complex bound poorly to gB in the envelope of HCMV virions, with only 0–2% of the gH/gL/gO complex or the pentameric complex found to bind to gB [42]. The function of the gB/gH/gL complex is currently unknown, although it is suggested that the gB/gH/gL complex stabilizes gB and keeps the gB in pre-fusion confirmation [42,53]. The identification of the gB/gH/gL complex makes it a unique vaccine candidate for HCMV, as the components of HCMV core fusion machinery—gB, gH, and gL—are required for HCMV fusion and enter into all the target cells. It also makes the combination of gB, gH, and gL a more attractive HCMV vaccine candidate. 

HCMV gH/gL are essential components of the gH/gL/gO complex, the pentameric complex, and the gB/gH/gL complex. An anti-gH/gL human monoclonal antibody MSL-109 recognizes HCMV protein complexes containing gH and blocks the infection of fibroblasts by laboratory and clinical HCMV strains [67]. More importantly, an alphavirus replicon particles (VRPs) vaccine platform expressing HCMV gH/gL elicited potent, broadly cross-reactive complement-independent neutralizing antibodies against HCMV [68]. Further, the neutralizing antibodies induced by gH/gL were significantly stronger and qualitatively different from those elicited by VRPs expressing HCMV gB (68). Although HCMV monomeric gH/gL could elicit a potent immune response, its immunogenicity could be significantly enhanced by a multimeric form [44,45,46]. We have previously produced EBV tetrameric gp350 and trimeric gH/gL, which induced up to 25-fold and 90-fold higher antigen-specific antibody responses respectively relative to their monomeric counterparts [43,47]. EBV tetrameric gp350 elicited 20-fold to 40-fold higher neutralizing antibodies compared to monomeric gp350 analyzed with a competitive neutralizing assay, and EBV trimeric gH/gL elicited fivefold higher neutralizing antibodies compared to EBV monomeric gH/gL analyzed with neutralizing assay for the prevention of EBV infection of a B lymphoma cell line [43,47]. In the current study, HCMV trimeric gH/gL induced up to 38-fold higher serum titers of gH/gL-specific IgG relative to monomeric gH/gL, and elicited approximately 10-fold higher complement-dependent and complement-independent HCMV-neutralizing antibodies measured using either epithelial cells or fibroblasts. As the combination of HCMV trimeric gB and monomeric gH/gL could elicit strong synergistic HCMV-neutralizing activity [41], whereas trimeric gH/gL could induce markedly higher gH/gL-specific IgG and HCMV-neutralizing antibodies compared to monomeric gH/gL, trimeric gH/gL can be used to replace monomeric gH/gL in HCMV vaccine studies. Trimeric HCMV gB in combination with trimeric gH/gL would be a novel promising HCMV vaccine candidate that could induce highly potent neutralizing activities for both fibroblast and epithelial cells. Non-neutralizing antibody functions such as antibody-dependent cell-mediated phagocytosis (ADCP) and antibody-dependent cellular cytotoxicity (ADCC) may play an important role in the prevention of HCMV infections [55,69,70]. The non-neutralizing antibody functions of the markedly higher anti-gH/gL antibodies induced by HCMV trimeric gH/gL should be determined, in addition to their potent neutralizing activity in future studies [55,69,70].

## 4. Materials and Methods

### 4.1. Cell Lines, HCMV Strains, and Reagents

The Chinese hamster ovary (CHO) cell line DG44 was purchased from Thermo Fisher Scientific (Waltham, MA), and maintained in CD DG44 medium. MRC-5 and ARPE-19 cell lines were purchased from ATCC, and cultured using EMEM or DMEM/F-12K medium respectively, both supplemented with 10% fetal bovine serum. HCMV strain AD169^wt131^ was provided by Xiao Wang and Haruhiko Murata (Food and Drug Administration) and strain AD169 was purchased from ATCC. HCMV strain AD169^wt131^ was propagated in ARPE-19 cells, and HCMV strain AD169 was propagated in MRC-5 cells. Monoclonal mouse IgG1 anti-gH antibody (0861) was purchased from Santa Cruz Biotechnology (Dallas, TX, USA). Horseradish peroxidase labeled anti-mouse antibody and goat anti-rabbit antibody were purchased from Thermo Fisher Scientific (Waltham, MA, USA).

### 4.2. Expression and Purification of HCMV Monomeric and Trimeric gH/gL Recombinant Proteins

The coding sequences for HCMV gH and gL were downloaded from NCBI, reference sequence # NC_006273.2, strain Merlin. For the trimeric gH/gL DNA expression construct, the signal peptide 1-30 of gL was replaced with an IgG κ leader sequence, and the gL sequence encoding amino acids (AA) 31-278 was linked to the gH sequence encoding AA 24-718, which was separated by a 15 amino acid linker (Gly_4_Ser)_3_ sequence. The foldon trimerization domain coding sequence derived from T4 phage fibritin was linked to the 3’ end of gH, followed by a His_6_ coding sequence (Figure 1C). DNA coding for the trimeric gH/gL was synthesized by Blue Heron Biotech (Bothell, WA, USA), cloned into pOptiVEV (Thermo Fisher Scientific, Waltham, MA, USA), and verified by sequencing. The HCMV monomeric gH/gL construct was made by the PCR amplification of HCMV timeric gH/gL with the foldon coding sequence deleted, cloned into pOptiVEV, and verified by sequencing. CHO cells were transfected with pOptiVEC-gH/gL monomeric or trimeric constructs using Free-style Max reagent (Thermo Fisher Scientific, Waltham, MA, USA), and selected with increasing concentrations of methotrexate up to 4 µM for optimal protein expression, followed by limiting dilution cloning. Stable CHO cell lines were cultured in a FiberCell bioreactor (FiberCell Systems, Frederick, MD, USA), and the supernatants were concentrated for affinity purification using a cobalt column (Thermo Fisher Scientific, Waltham, MA) following the manufacturer’s instructions, and further purified using size exclusion chromatography on a Superdex 200 column (GE Lifesciences, Pittsburgh, PA, USA).

### 4.3. Western Blot Analysis

Purified proteins were analyzed by electrophoresis on 3–8% NuPAGE Tris-Acetate Mini-Gels under reducing conditions or modified non-reducing conditions. Under reducing conditions, purified HCMV monomeric or trimeric gH/gL recombinant proteins were boiled for 10 min in lithium dodecyl sulfate (LDS) loading buffer containing 50 mM of dithiothreitol (DTT), and resolved on 3–8% PAGE in SDS running buffer. For modified non-reducing conditions, protein samples were mixed with LDS loading buffer without DTT, and resolved on 3–8% PAGE in tris-glycine native running buffer (Thermo Fisher Scientific, Waltham, WA, USA). Proteins were transferred to membranes, and probed using a mouse monoclonal anti-gH antibody (0861) from Santa Cruz Biotechnology (Dallas, TX, USA), followed by horseradish peroxidase labeled anti-mouse antibody from Thermo Fisher Scientific (Waltham, MA, USA). Membranes were then incubated with SuperSignal West Pico chemiluminescent substrate with a signal captured on X-ray film.

### 4.4. Rabbit Immunization

Groups of five New Zealand white rabbits, 12–15 weeks old, were immunized subcutaneously with 25 µg of of HCMV monomeric or trimeric gH/gL recombinant proteins adsorbed to aluminum hydroxide and mixed with 50 µg of a 12-mer phosphorothioate-modified CpG-ODN (tcataacgttcc) optimized for rabbits (48). Rabbits were immunized on days 0, 21, and 42, and serum samples were taken before the initial immunization and 10 days following each immunization. These studies were conducted in accordance with the Guide for Care and Use of Laboratory Animals (Institute of Laboratory Animal Resources, NRC), and were approved by the USUHS Institutional Animal Care and Use Committee (USUHS PAT 18-186, 2 January 2018).

### 4.5. Measurement of Serum Titers of HCMV gH/gL-Specific IgG by ELISA

Immulon 4 ELISA plates were coated overnight with 5 µg/mL of HCMV monomeric gH/gL recombinant protein in PBS at 4 °C, and blocked with 1% bovine serum albumin (BSA) in PBS. Threefold serial dilutions of serum samples in 1% BSA–PBS were tadded and incubated overnight at 4 °C, followed by washing with 0.1% Tween-20 in PBS. Alkaline phosphatase-conjugated polyclonal goat anti-rabbit IgG (Southern Biotechnology, Birmingham, AL, USA) in 1% BSA–PBS was added, and plates were incubated at 37 °C for 1 h. Plates were washed with 0.1% Tween-20 in PBS, and substrate (p-nitrophenyl phosphate, disodium; Sigma-Aldrich, St. Louis, MO, USA) was added at 1 mg/mL in tris-HCl magnesium-sulfate (TM) buffer for color development. Color was read at an absorbance of 405 nm on a Multiskan Ascent ELISA reader. 

### 4.6. Determination of Serum HCMV-Neutralizing Titers

Day 52 sera from rabbits immunized with HCMV monomeric or trimeric gH/gL recombinant proteins were either heat inactivated at 56 °C for 30 min to eliminate complement activity or not heat-treated. Serum HCMV-neutralizing antibody titers were determined using an ELISpot assay as described [71]. Each serum sample was initially diluted at a ratio of 1:10 and prepared as 1:2 serial dilutions in cell culture medium in triplicates. Each dilution was mixed with an equal volume of culture medium containing 4000 pfu/ml HCMV strain AD169^wt131^ [33,35,36], incubated for 4 h at 37 °C, and then added to the wells of 96-well plates containing MRC-5 or ARPE-19 monolayers and cultured overnight at 37 °C in 5% CO_2_. Cells were fixed with absolute ethanol, rehydrated, and blocked with 5% normal horse serum in PBS, followed by incubation with anti-IE1 monoclonal antibody MAB810 (Millipore, Burlington, MA, USA), goat anti-mouse secondary antibody (Jackson ImmunoResearch Labs, West Grove, PA, USA) each for 1 h, and VECTASTAIN ABC reagent (Vector Labs, Burlingame, CA, USA) for 30 minutes. Plates were washed 3x with 0.1% Tween 20 in PBS between each step, and TMB substrate (Mabtech, Inc., Cincinnati, OH, USA) was added for color development. The plates were scanned and analyzed using a CTL-ImmunoSpot® S6 Micro Analyzer. Fifty percent inhibitory concentration (IC50) values and standard errors of the means were calculated using GraphPad Prism7 software by plotting the means of triplicate values for each serum dilution against the log serum dilution, calculating the best fit four-parameter equation for the data, and interpolating the inverse serum dilution at the mid-point of the curve as the IC50 neutralizing titer.

### 4.7. Statistics

All the studies were repeated at least once for reproducibility. Serum titers of antigen-specific antibodies and HCMV-neutralizing activities were expressed as geometric means +/− the standard error of the mean, with significance determined by a two-tailed Student’s *t*-test (*p* < 0.05 is considered significant).

## 5. Conclusions

Vaccine development against HCMV is a major public health priority, as congenital HCMV infection and HCMV infection of the immunosuppressed patients cause significant morbidity and mortality. Natural immunity against HCMV infection is protective, but not complete. Subunit vaccine candidates such as recombinant HCMV envelope proteins have the potential to elicit immune responses that are quantitatively or qualitatively different from those induced by HCMV during natural infection. HCMV protein antigens expressing native conformational epitopes could elicit optimal immune responses, and the use of a combination of HCMV recombinant proteins such as the components of the core fusion machinery (trimeric gB and trimeric gH/gL) is a safe and efficient approach for HCMV vaccine development that could potentially provide superior protection over natural immunity. 

## Footnotes, Mandatory Disclaimer

The opinions expressed herein are those of the authors and are not necessarily representative of those of the Uniformed Services University of the Health Sciences (USUHS), the Department of Defense (DOD), or the United States Army, Navy or Air Force.
